# Clinical impact of low serum free T4 in patients with non-small cell lung cancer treated with nivolumab

**DOI:** 10.1038/s41598-019-53327-7

**Published:** 2019-11-19

**Authors:** Tomoko Yamamoto Funazo, Takashi Nomizo, Hiroaki Ozasa, Takahiro Tsuji, Yuto Yasuda, Hironori Yoshida, Yuichi Sakamori, Hiroki Nagai, Toyohiro Hirai, Young Hak Kim

**Affiliations:** Department of Respiratory Medicine, Graduate School of Medicine, Kyoto University, 54, Shogoin-kawaharacho, Sakyo-ku, Kyoto, 606-8507 Japan

**Keywords:** Cancer immunotherapy, Tumour biomarkers, Haplotypes

## Abstract

Nivolumab improves the prognosis of non-small cell lung cancer (NSCLC) but can cause immune-related adverse events (irAEs). Reports have indicated longer progression-free survivals (PFSs) in patients with irAEs than in those without irAEs. We reported associations between programmed death ligand-1 (PD-L1) single nucleotide polymorphisms (SNPs) and PFS after nivolumab treatment. We hypothesized that adverse events might be associated with the SNPs of PD-L1. We analyzed data from 111 patients with NSCLC treated with nivolumab. The response rate was 14%, and the median PFS was 68 days. We found patients with the adverse event of low free tetraiodothyronine (fT4) had significantly longer PFSs than those without low fT4 (not reached vs 67 days; hazard ratio [HR], 0.297; P = 0.010). Moreover, median overall survival was longer in patients with low fT4 than those without low fT4 (not reached vs 556 days, HR, 0.139; P = 0.020). Patients with the T allele of rs1411262 (P = 0.0073) and with the A allele of rs822339 (P = 0.0204) developed low fT4. Patients with the T/T genotype had longer PFSs than with those with the C/T and C/C genotypes of rs1411262 (165 vs. 67 days, HR, 1.65; P = 0.040), and those with the A/A genotype had longer PFSs than those with the A/G and G/G genotypes of rs822339 (182 vs. 67 days, HR, 1.76; P = 0.025). In the patients with advanced NSCLC, low fT4 after nivolumab treatment was associated with significantly longer PFSs. The SNPs of PD-L1 may be associated with the adverse events of nivolumab.

## Introduction

Nivolumab is one of the immune-checkpoint inhibitors (ICIs) that inhibits the programmed death 1 (PD-1) and the programmed death ligand 1 (PD-L1) pathways and releases effector T cells as a defense against tumor cells. Nivolumab dramatically improved the prognosis of patients with non-small cell lung cancer (NSCLC) with clinical trials showing longer overall survivals (OS) in the nivolumab-treated than in the docetaxel-treated patients with advanced NSCLC^1,2^. In addition, some patients had responses lasting more than 2 years^3^; although this response rate can be as low as 20%. Biomarkers including PD-L1 expression in the tumor cells^4^, the presence of tumor-infiltrating lymphocytes at the invasive tumor margin^5^, and the nonsynonymous mutation burden frequency in the tumor cells^6^ have been shown to predict the effect of nivolumab. Although the PD-L1 tumor cell expression has been studied as a biomarker in many clinical trials, its prediction accuracy is limited^4,7^

Nivolumab treatment also results in a unique side-effect profile caused by the action of effector T cells against self-antigens, described as immune-related adverse events (irAEs), and includes rashes, colitis, diarrhea, thyroiditis, hypophysitis, hepatitis, pancreatitis, iridocyclitis, lymphadenopathy, neuropathies, and nephritis^8,9^. Previous reports have suggested that patients with ICI-induced irAEs have longer PFS and OS than those without irAEs^10–12^. The irAEs could be the product of the responses to immune system activation by ICIs.

We have reported an association between single nucleotide polymorphisms (SNPs) of PD-L1 and the response to nivolumab^13^. A similar report indicated an association between SNPs and the response to other ICIs^14^. Moreover, other studies have also indicated an association between the SNPs of PD-1 and PD-L1 and autoimmune diseases such as systemic lupus erythematosus^15^, type 1 diabetes^16^, and Addison’s disease, and Graves’ disease^17,18^. According to these reports, the SNPs of PD-L1 may be associated with the functioning of the PD-1 and PD-L1 pathway. However, the association between SNPs and the adverse events of ICIs remains unclear.

We hypothesized that the adverse events of nivolumab in patients with NSCLC might be associated with the treatment response and with PD-1/PD-L1 SNPs. We retrospectively analyzed data from patients with advanced NSCLC treated with nivolumab to assess the association between adverse events and response, and the association between adverse events and PD-1/PD-L1 SNPs.

## Results

### Patients’ characteristics

Table 1 lists patients’ characteristics. The median age was 68 years with a wide range from 33 to 85 years, and 73 patients (66%) were men. Most patients (91%) had an ECOG PS of 0 or 1. Almost three-quarters of patients were diagnosed as having adenocarcinoma and one-fifth of patients as having squamous-cell carcinoma. Forty-eight patients (43%) received nivolumab as the second-line treatment, and 63 (57%) received it as the third-line or later treatment.

**Table 1:** Patients’ characteristics.

		Total
		N = 111
Sex	Male/female	73/38
Age	Median (range)	68 (33–85)
Smoking status	Never/ever	33/78
Numbers of line	2nd/≧3rd	48/63
ECOG PS	0 or 1/2	100/11
Histology	Adenocarcinoma Squamous Other LCNEC	81 21 7 2
	EGFR mutated ALK rearranged	29 4

### Clinical outcomes

Among the 111 patients, none achieved a complete response, 16 (14%) had a partial response (PR), 42 (38%) had stable disease (SD), and 53 (48%) had PD. The median PFS in all patients was 68 days (95% confidence interval [CI], 58–101 days) and was similar to that reported in a large clinical trials of nivolumab in patients with NSCLC^1,2^.

### Adverse events

From 111 patients, 108 patients with follow-up blood exam were analyzed for adverse events. Table 2 lists the adverse events that occurred during nivolumab treatment in the participants. The most frequent adverse event was malaise, but grade 3 treatment-related adverse events included gastritis and headache. We found no grade 4 or 5 adverse events. The profile and frequency of adverse events in this study were similar to those reported for clinical trials of nivolumab in patients with NSCLC^19,20^.

**Table 2:** List of adverse events. Adverse events that occurred during nivolumab treatment in the participants.

	grade 1	grade 2	grade 3	grade 4	Incidence
Malaise	19	2	0	0	20%
Liver dysfunction	21	0	0	0	19%
Thyroid dysfunction	14	6	0	0	18%
Anorexia	11	4	0	0	14%
Pneumonitis	8	1	0	0	10%
Rash	9	0	0	0	8%
Fever	4	0	0	0	4%
Oral ulcer	1	0	0	0	1%
Gastritis	0	0	1	0	1%
Headache	0	0	1	0	1%
Hypophysitis	0	1	0	0	1%
Infusion reaction	0	1	0	0	1%

### Association between PFS and adverse events

**Figure 1:**
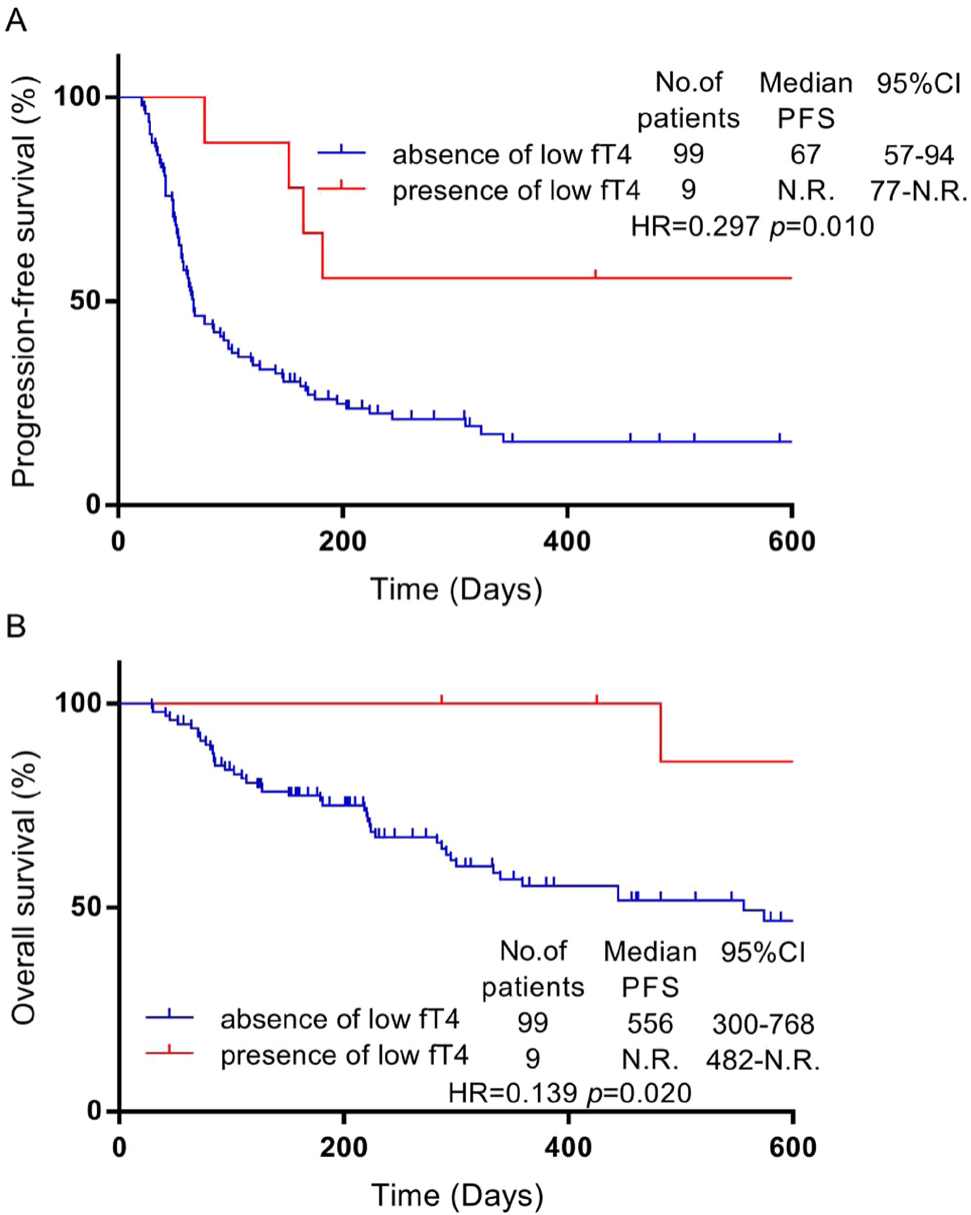
PFS after the administration of nivolumab. The Kaplan–Meier curves for progression-free survival (**A**) and overall survival (**B**) are presented. The red line represents the presence of low fT4 level, and the blue line represents the absence of low fT4. N.R. denotes not reached.

The associations between PFS and each adverse event were analyzed for 108 patients. The median PFS was significantly longer in patients with low free tetraiodothyronine (fT4) level than in those without low fT4 (not reached vs. 67 days; hazard ratio [HR]: 0.297; P = 0.010; Fig. 1A), and the median OS was longer in patients with low fT4 level than in those without low fT4 (not reached vs. 556 days; HR: 0.139; P = 0.020; Fig. 1B). We defined low fT4 levels as those below the facility standard. We found no significant associations between low fT4 and other clinical variables including age, gender, smoking status, ECOG PS, number of chemotherapy cycles, histological findings, and EGFR mutation status (Supplementary Table S1). We found no significant associations between other adverse events and the PFS (Supplementary Fig. S1).

### Association between SNPs and Adverse events

We evaluated the prevalences of PD-1/PD-L1 SNPs and compared them between patients with and without individual adverse events. We found a significant association between a low fT4 and the SNPs of PD-L1 (Table 3). The patients with the T allele of rs1411262 and the A allele of rs822339 were more likely to present low fT4 than patients with the C allele of rs1411262 or the G allele of rs82239. We found no significant associations between other adverse events and SNPs (Supplementary Table S2). In this study, rs1411262 and rs822339 were in linkage disequilibrium relationship (Supplementary Fig. S2).

**Table 3:** Association between PD-1/PD-L1 SNPs and adverse events.

SNPs			low fT4		P value
			presence (n = 9)	absence (n = 99)	
rs1411262 (PD-L1)	Genotype	CC	0	26	**0.0073**
		CT	3	48	
		TT	6	25	
	Allele	CC + CT	3	74	**0.0016**
		TT	6	25	
rs822339 (PD-L1)	Genotype	AA	5	24	**0.0204**
		AG	4	49	
		GG	0	26	
	Allele	AA	5	24	0.057
		AG + GG	4	75	
rs2282055 (PD-L1)	Genotype	GG	5	31	0.179
		GT	3	48	
		TT	1	20	
	Allele	GG + GT	8	79	1.000
		TT	1	20	
rs4143815 (PD-L1)	Genotype	CC	3	24	0.281
		CG	5	46	
		GG	1	29	
	Allele	CC	3	24	0.688
		CG + GG	6	75	
rs2890658 (PD-L1)	Genotype	AA	0	4	0.796
		AC	4	31	
		CC	5	64	
	Allele	AA	0	4	1.000
		AC + CC	9	95	
rs2227981 (PD-1)	Genotype	AA	0	6	0.699
		AG	4	40	
		GG	5	53	
	Allele	AA	0	6	1.000
		AG + GG	9	93	
rs2227982 (PD-1)	Genotype	AA	2	22	0.838
		AG	4	49	
		GG	3	28	
	Allele	AA + AG	6	71	0.714
		GG	3	28	

Frequency of alleles and distribution of genotypes of PD-L1/ PD-1 SNPs in patients with low free T4 (n = 9) and those without low free T4 (n = 99). P-values are as shown in the table, and significant results are indicated in bold.

### Association of SNPs and PFS

**Figure 2:**
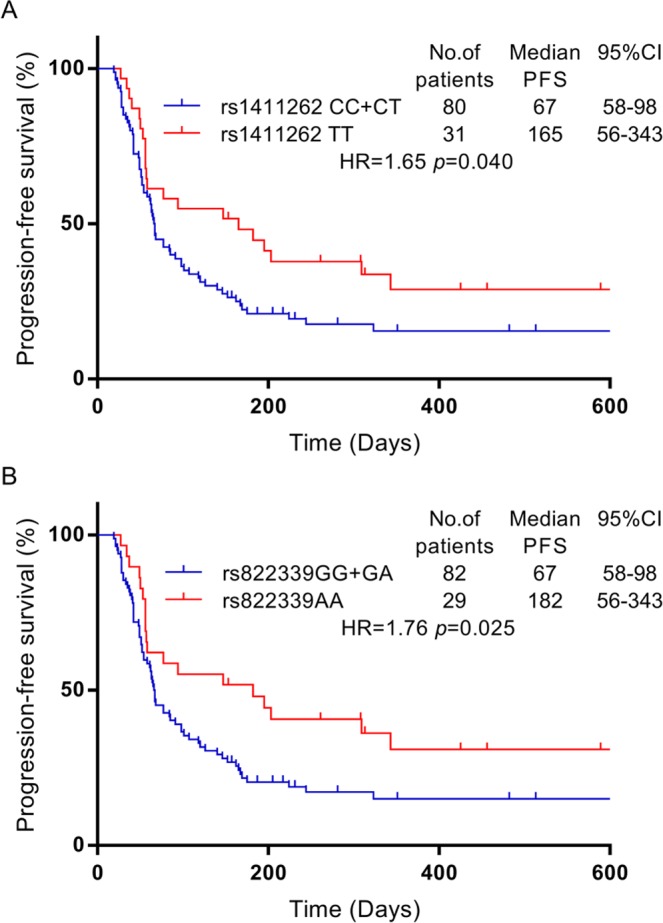
PFS after nivolumab administration shown by a Kaplan–Meier curve. The red line represents the C/C and C/T genotypes of rs1411262, and the blue line represents the T/T genotype of rs1411262 (**A**). The red line represents the G/G and A/G genotypes of s822339, and the blue line represents the A/A genotype of rs822339 (**B**).

We examined the possible associations between PFS and PD-1/PD-L1 SNPs. The T/T genotype of rs1411262 was associated with longer PFS than the C/T and C/C genotypes of rs1411262 (165 vs.67 days, respectively; HR, 1.65; P = 0.040; Fig. 2A) and the A/A genotype of rs822339 was also significantly associated with longer PFS than the A/G and G/G genotypes of rs822339 (182 vs.67 days, respectively; HR, 1.76; P = 0.025; Fig. 2B).

### Patients with low fT4

Supplementary Fig. S3 and Supplementary Table S3 show the characteristics of patients with low fT4. The median duration of time from the administration of nivolumab to the onset of thyroid dysfunction was 48 days. Three patients had PR, 6 patients had SD, but none had PD. Five patients discontinued the administration of nivolumab due to adverse events. Of the 9 patients, 5 patients have maintained tumor shrinkage until the publication of this paper, 2 had relapsed and 2 switched chemotherapies before disease progression at the discretion of their physicians.

## Discussion

Here, we showed that patients with low fT4 had longer PFS than those without low fT4. We found associations between the occurrence of low fT4 and SNPs of PD-L1; rs1411262 and 822339. Patients with the T/T genotype of rs1411262 and the A/A genotype of rs822339 were susceptible to low T4 and had longer PFSs. This is the first report showing an association between SNPs of PD-L1 and the occurrence of adverse events.

We focused on the low fT4 level adverse event. The definitions of thyroid dysfunction in the literature do not include the criteria for thyroid dysfunction caused by ICIs. In general medical settings, thyroid dysfunction is often diagnosed based on a high TSH level, low fT4 level, and low free triiodothyronine (fT3) level. In the present study, 7 patients had a TSH level ≥10 mIU/l among the 9 cases that showed low fT4 levels (Supplementary Table S3). High TSH levels are commonly attributed to low fT4 level feedback. The influence of nivolumab on this feedback is unclear. In addition, an elevated TSH level may not be confirmed based on the timing of blood examination. Reduction of fT3 level can occur in low T3 syndrome, a nonthyroidal illness associated with critical illness, including cancer^21,22^. We believe that fT4 level is an index that can be used for the evaluation of thyroid dysfunction as an adverse event of nivolumab.

The thyroid dysfunction caused by nivolumab has been considered due to a direct attack by effector T cells against tumor-associated self-antigens^19^. The hypothesis on effector T cells attacking tumor as well as thyroid cells could explain why patients developing thyroid dysfunction tend to have a more therapeutic response to ICI treatment. Tanaka *et al*. found that one of three patients who developed thyroid dysfunction achieved complete remission after nivolumab treatment^23^. This finding was similar to our results showing that patients with low fT4 had PR or SD. Moreover, of our nine patients who developed low fT4; two have maintained tumor shrinkage even after treatment interruption because of adverse events (Supplementary Fig. S3).

Although no reports have ascribed a role to PD-L1 in the onset of thyroid dysfunction including Grave’s disease and Hashimoto’s thyroiditis, PD-L1 deficiency has been associated with the onset and exacerbation of various autoimmune diseases, including type 1 diabetes^24,25^, rheumatoid arthritis^26,27^, and ankylosing spondylitis^28^. SNPs of PD-L1 have also been associated with autoimmune diseases: Mitchell *et al*. showed that the frequencies of the C allele of rs1411262 and the G allele of rs822339 were higher in patients with Addison’s disease and Graves’ disease than in controls^18^. Yang *et al*. reported that the C allele of rs822336 was more frequent in patients having ankylosing spondylitis than in controls^29^, and Pizarro *et al*. reported that the G allele of rs4143815 and the G allele of rs2297137 were higher in patients with type 1 diabetes than in healthy controls^16^. Interestingly, Shi *et al*. showed that the G/G genotype of rs4143815 was associated with lower PD-L1 expression on hepatic dendritic cells after IFN-γ stimulation^30^. These reports indicate that SNPs of PD-L1 and expression of PD-L1 may be related. The C allele of rs1411262 and the G allele of rs822339 are in linkage disequilibrium with the C allele of rs822336, the G allele of rs4143815, and the G allele of rs2297137 according to the LD link database (Supplementary Table S4). Therefore, rs1411262 and rs822339 may also be involved in the protein expression of PD-L1 and may affect PD-L1 function. In this study, we evaluated seven SNPs with a relatively even distribution of allele frequencies in Japanese patients and reported the association with autoimmune diseases. We speculate that patients with the T allele of rs1411262 and the A allele of rs822339 have a low frequency of fT4 during nivolumab treatment due to activated immune cells that may attack the thyroid gland.

Our study had some limitations. First, it was a retrospective cohort study with a small sample size. The median follow-up period was about 12 months, and the OS was immature. Long-term adverse events might not have been fully evaluated. Large-sized studies may confirm the relevance of PD-L1 SNPs to other clinically important adverse events, including pneumonitis. We are planning future studies to evaluate the relevance of the adverse events of nivolumab and PD-1/PD-L1 SNPs (UMIN000033839). Second, PD-L1 expression has not been completely analyzed. Of the nine patients who presented with low fT4 level, one tested negative for PD-L1 (tumor proportion score <1%), and the PD-L1 status of the other patients was not assessed (Supplementary Table S3). PD-L1 expression was evaluated only in 49 (45%) patients (Supplementary Fig. S4). Thus, the relationship between TPS and adverse events must be assessed in future studies. Last, we selected seven PD-1/PD-L1 polymorphisms that were considered functional based on previous reports; however, other more efficiently predictable SNPs may exist.

In conclusion, we found that PFS is longer in patients with low fT4 than in those without low fT4 following treatment with nivolumab for advanced NSCLC. The T allele of rs1411262 and A allele of rs822339 were significantly associated with low fT4 and the T/T genotype of rs1411262 and A/A genotype of rs822339 were associated with longer PFS. SNPs of PD-L1 may lead to the activation of the PD-1 and PD-L1 pathways that attack both tumor tissue and normal tissue. This is the first report to show an association between adverse events and SNPs of PD-L1 in patients with NSCLC treated with nivolumab.

## Patients and Methods

### Patients

**Figure 3:**
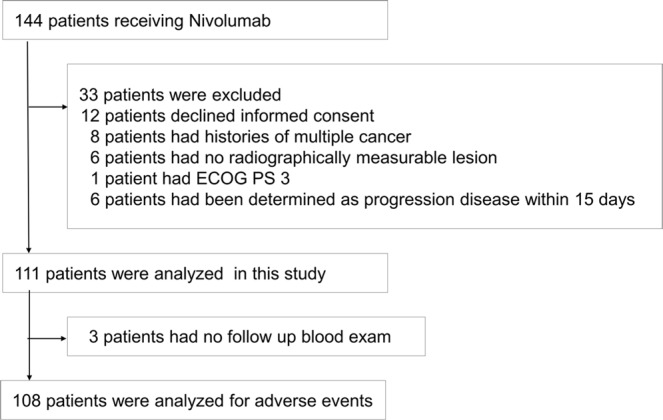
Flow of Patients in this study.

Between January 2016 and December 2017, a total of 144 consecutive patients were diagnosed with non-small cell lung cancer and were treated with nivolumab at the Kyoto University Hospital. Of these patients, 111 patients were analyzed for association between PFS and PD-1/PD-L1 SNPs. We excluded patients who declined informed consents, patients with multiple cancer histories, patients without radiographically measurable lesions, patients with Eastern Cooperative Oncology Group (ECOG) Performance Status (PS) 3, and patients diagnosed with disease progression within 15 days from the first dose (Fig. 3). From these patients, 108 patients with follow-up blood exam were analyzed for adverse events. We collected 10-mL peripheral blood samples from each patient to examine genotypes. We followed the guidelines in the Declaration of Helsinki to conduct our study. The Review Board of the Kyoto University Hospital approved the study’s protocol (certification number: G0788). All participants provided written informed consents.

### Genotyping and SNP selection

Genomic DNA was extracted from peripheral blood leukocytes using Gene Prep Star NA-480 (Kurabo, Osaka, Japan). Genotyping was performed using the TaqMan® genotyping assay (Applied Biosystems, Foster City, CA. USA) and analyzed using the Applied Biosystems 7300 Real-Time polymerase chain reaction (PCR) System (Applied Biosystems). The PCR solution contained a 1-µL DNA sample, 12.5 µL of 2X TaqMan Universal PCR Master Mix, 0.3125 µL of primer probe mix, and 11.2 µL of nuclease-free water. After baseline fluorescence measurements at 25 °C, the following PCR protocol was performed: The samples were incubated for 10 min at 95°C, subjected to 40 cycles of denaturing at 92 °C for 15 s, and annealed and extended at 60 °C for 1 min, with a final measurement of fluorescence at 60 °C. We selected SNPs associated with autoimmune disease or putative functional effects^15–18^.

### Evaluation of nivolumab efficacy and adverse events

We extracted clinical features and treatment histories from medical records updated until April 2018. PD-L1 expression of tumor was evaluated in pretreatment tumor obtained from biopsy or surgery using PD-L1 immunohistochemistry 22C3 pharmDx assay at a commercial clinical laboratory in SRL, Inc. (Tokyo, Japan). Nivolumab was administered intravenously to patients at doses of 3 mg/kg every two weeks and until evaluation of progression disease (PD) or unacceptable adverse events appeared. Radiographic imaging was performed every 6 to 8 weeks. The responses were evaluated separately by two researchers according to the Response Evaluation Criteria in Solid Tumors (RECIST) (version 1.1). PFSs were measured from the start of nivolumab administration until the day of RECIST PD. We censored data from patients with no recorded clinical or radiological disease progression on the day of the last follow-up.

One of the investigators assessed the adverse events according to the Common Terminology Criteria for Adverse Events (version 4.0). Blood examinations were carried out every 2 weeks, and thyroid function tests (TSH, free T3, and free T4 (fT4) levels) were performed every 4 to 6 weeks.

### Statistical analysis

We calculated the prevalences of genotypes and alleles and compared them between patients with adverse events and those without adverse events. We evaluated the statistical significances of the associations between the frequency of adverse events and genotypes using Fisher’s exact test or the Cochrane–Armitage test, as appropriate. We calculated survival curves using the Kaplan–Meier method and used the log-rank test to evaluate the statistical significance of the associations between PFS and genotypes, and between PFS and adverse events. We performed all of the above statistical analyses using the JMP Pro statistical software version 12.1.0 (SAS Institute, Cary, NC, USA). We used the HaploView software (Broad Institute, Cambridge, MA, USA) to estimate the linkage disequilibrium, expressed as D’ and r^2^.

## Supplementary information

Table S1-4, Figure S1-4
