# Supplementary material for: Clinical impact of low serum free T4 in patients with non-small cell lung cancer treated with nivolumab
Source: Sci Rep. 2019 Nov 19;9:17085. doi: 10.1038/s41598-019-53327-7 (PMC6864095; doi:10.1038/s41598-019-53327-7)
Supplement: Supplementary file 1 — Table S1-4, Figure S1-4 [file 41598_2019_53327_MOESM1_ESM.pdf]

Clinical impact of low serum free T4 in patients with non-small cell lung cancer treated with nivolumab

Tomoko Yamamoto Funazo, Takashi Nomizo, Hiroaki Ozasa*, Takahiro Tsuji, Yuto Yasuda, Hironori Yoshida, Yuichi Sakamori, Hiroki Nagai, Toyohiro Hirai, Young Hak Kim

Department of Respiratory Medicine, Graduate School of Medicine, Kyoto University, 54, Shogoin-kawaharacho, Sakyo-ku, Kyoto 606-8507, Japan

***Corresponding author: Hiroaki Ozasa,** Department of Respiratory Medicine, Graduate School of Medicine, Kyoto University, 54, Shogoin-kawaharacho, Sakyo-ku, Kyoto 606-8507, Japan. Tel: (+81) 75 751 3830. Fax: (+81) 75 751 4643.

Email: ozahiro@kuhp.kyoto-u.ac.jp

Table S1. Characteristics of all 108 patients and patients with low free T4. Calculated P values are shown in the right column for comparison of clinical characteristics between 108 patients evaluated for adverse events and 9 patients with low fT4.

		absence of low Free T4 N = 108	presence of low Free T4 N = 9	P value
Sex	Male/female	72/36	6/3	1.000
Age	Median (range)	68 (33-85)	69 (52-83)	
Smoking status	Never/ever	33/75	2/7	0.719
Numbers of line	2nd/ ≧3rd	46/62	4/5	1.000
ECOG PS	0 or 1/2	99/9	9/0	1.000
Histology	Adenocarcinoma<br>Squamous<br>Other<br>LCNEC	80<br>21<br>5<br>2	5<br>2<br>2<br>0	
	EGFR mutated<br>ALK rearranged	28<br>4	1<br>0	0.441<br>1.000

Table S2. Association between PD-1/PD-L1 SNPs and adverse events. Frequency of alleles and distribution of genotypes of PD-L1/PD-1 SNPs in patients with adverse events and those without adverse events.

SNPs			liver dysfunction		P value	rash		P value	fever		P value
			presence (n = 21)	absence (n = 87)		presence (n = 9)	absence (n = 99)		presence (n = 4)	absence (n = 104)	
rs1411262 (PD-L1)	Genotype	CC	6	20	0.319	3	23	0.496	1	25	0.897
		CT	11	40		4	47		2	49	
		TT	4	27		2	29		1	30	
	Allele	CC+CT	17	60	0.420	7	70	1.000	3	74	1.000
		TT	4	27		2	29		1	30	
rs822339 (PD-L1)	Genotype	AA	4	25	0.378	2	27	0.542	1	28	0.937
		AG	11	42		4	49		2	51	
		GG	6	20		3	23		1	25	
	Allele	AA	4	25	0.426	2	27	1.000	1	28	1.000
		AG+GG	17	62		7	72		3	76	
rs2282055 (PD-L1)	Genotype	GG	5	31	0.320	2	34	0.113	1	35	0.691
		GT	11	40		3	48		2	49	
		TT	5	16		4	17		1	20	
	Allele	GG+GT	16	71	0.551	5	72	0.070	3	84	1.000
		TT	5	16		4	17		1	20	
rs4143815 (PD-L1)	Genotype	CC	2	25	0.070	1	26	0.187	1	26	0.938
		CG	11	40		4	47		2	49	
		GG	8	22		4	26		1	29	
	Allele	CC	2	25	0.092	1	26	0.445	1	26	1.000
		CG+GG	19	62		8	73		3	78	
rs2890658 (PD-L1)	Genotype	AA	1	3	0.477	0	4	0.717	0	4	0.711
		AC	8	27		3	32		2	33	
		CC	12	57		6	63		2	67	
	Allele	AA	1	3	1.000	0	4	1.000	0	4	1.000
		AC+CC	20	84		9	95		4	100	
rs2227981 (PD-1)	Genotype	AA	1	5	0.653	0	6	0.699	0	6	0.950
		AG	10	34		4	40		2	42	
		GG	10	48		5	53		2	56	
	Allele	AA	1	5	1.000	0	6	1.000	0	6	1.000
		AG+GG	20	82		9	93		4	98	
rs2227982 (PD-1)	Genotype	AA	6	18	0.250	1	23	0.838	2	22	0.105
		AG	11	42		6	47		2	51	
		GG	4	27		2	29		0	31	
	Allele	AA+AG	17	60	0.420	7	70	1.000	4	73	0.323
		GG	4	27		2	29		0	31	

Table S3. Characteristics of patients with low free T4 level. The number of patients is consistent with that presented in Supplementary Figure S3. M, male; F, female; PS, performance status; adeno, adenocarcinoma; Sq, squamous cell carcinoma; NSCLC, non-small cell carcinoma; TPS, Tumor Proportion Score

No. of Patient	rs1411262/ rs822339	age	sex	smoking status	No. of treatment line	PS	Histology	EGFR mutation	TPS	Response	T3 level	TSH level
1	TT/AA	72	M	ex	8	0	Adeno	negative		PR	normal	high
2	TT/AA	63	M	current	3	1	NSCLC	exon18		PR	low	normal
3	CT/AG	52	M	current	3	1	Adeno	negative		PR	high	high
4	TT/AA	63	F	ex	3	1	Adeno	negative		SD	low	low
5	TT/AA	69	M	ex	1	1	Sq	negative		SD	low	high
6	TT/AA	78	F	never	3	1	NSCLC	negative		SD	low	high
7	CT/AG	83	M	ex	1	0	Adeno	negative	< 1%	SD	low	high
8	CT/AG	70	M	ex	2	0	Sq	negative		SD	high	high
9	CT/AG	62	F	never	2	0	Adeno	negative		SD	low	high

Table S4. Linkage disequilibrium (absolute D' value) among SNPs of PD-L1 in East Asian patients according to the LD link database.

SNPs	rs822336	rs822339	rs1411262	rs2297137	rs4143815
rs822339	0.979	1.0	0.976	0.587	0.376
rs1411262	0.965	0.976	1.0	0.598	0.395

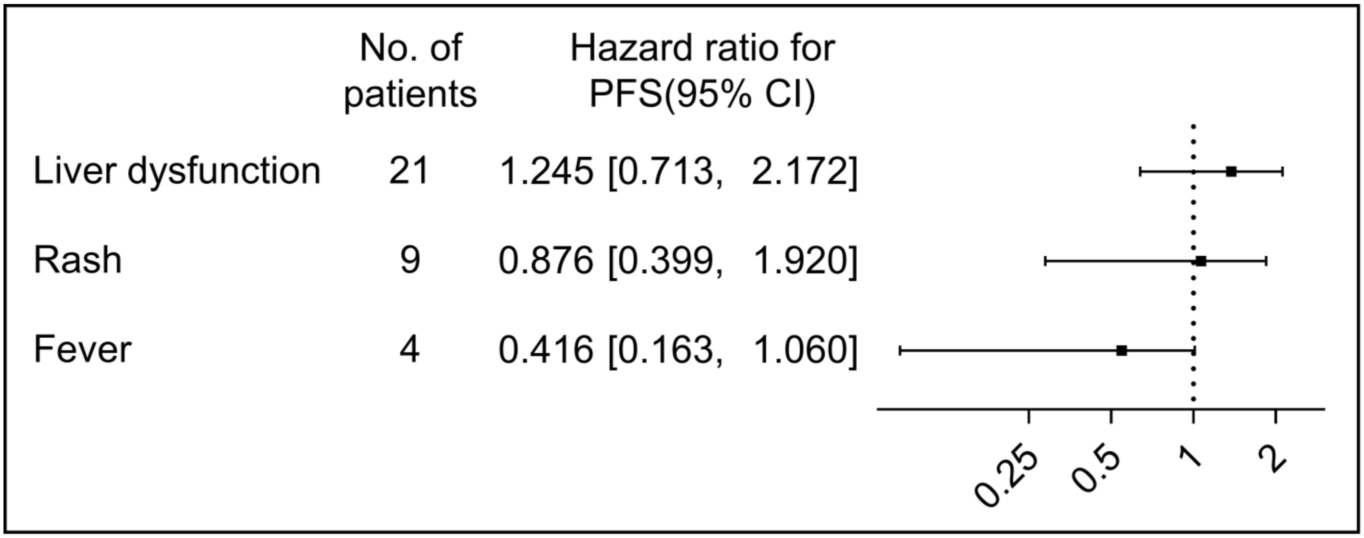

Figure S1. Progression-free survival (PFS) for patients with adverse events compared with those without adverse events. Forest plot showing the comparison between PFS for patients with and without adverse events.

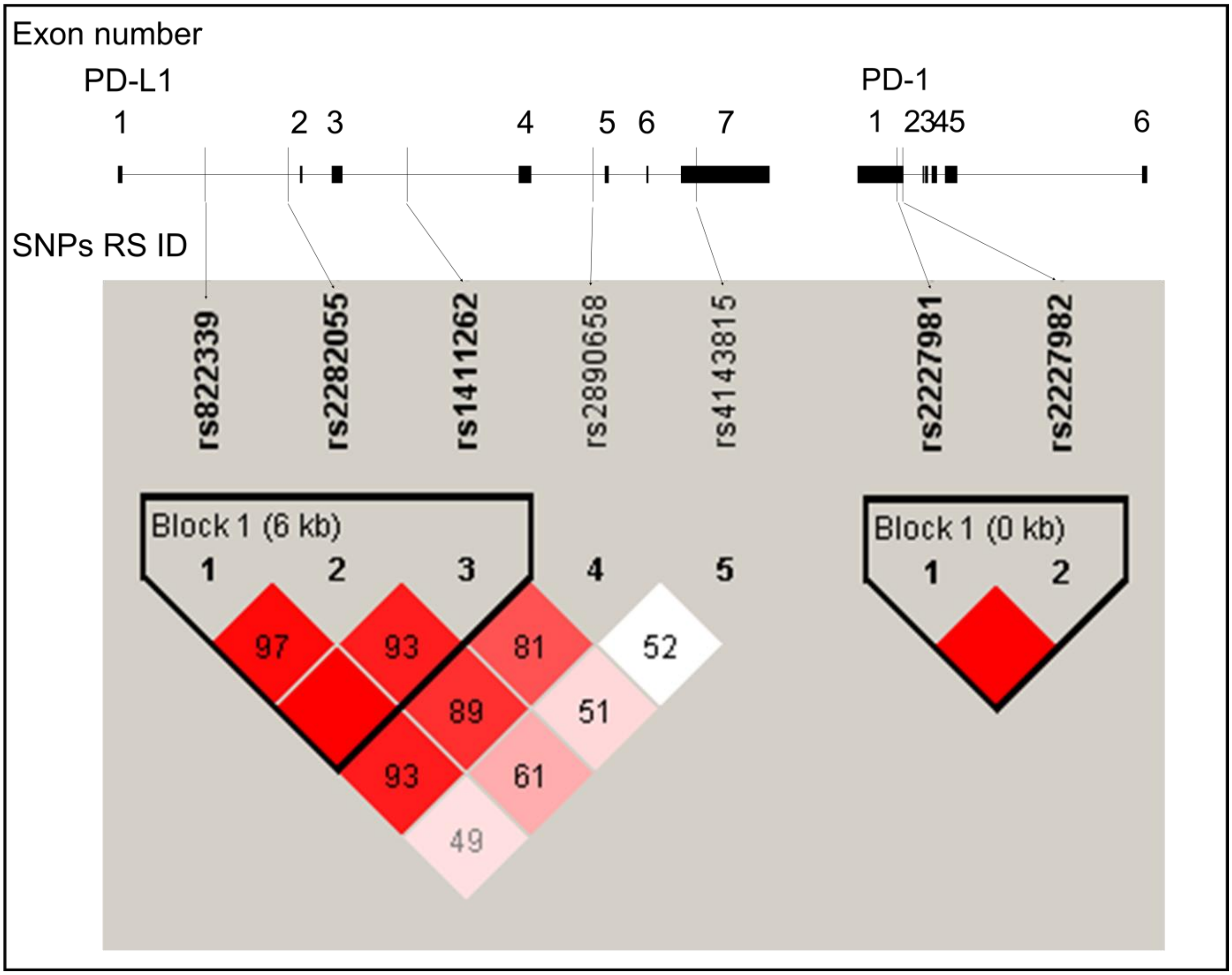

Figure S2. Genomic organization and linkage disequilibrium at the PD-L1 (left) and PD-1 locus (right). The upper panel represents a physical map of the genomic organization of the PD-L1 and PD-1 loci. Exons are numbered and indicated by black boxes. Numbers inside the squares represent the D' values expressed as percentages. Squares without numbers represent D' values of 1.0, indicating complete linkage disequilibrium.

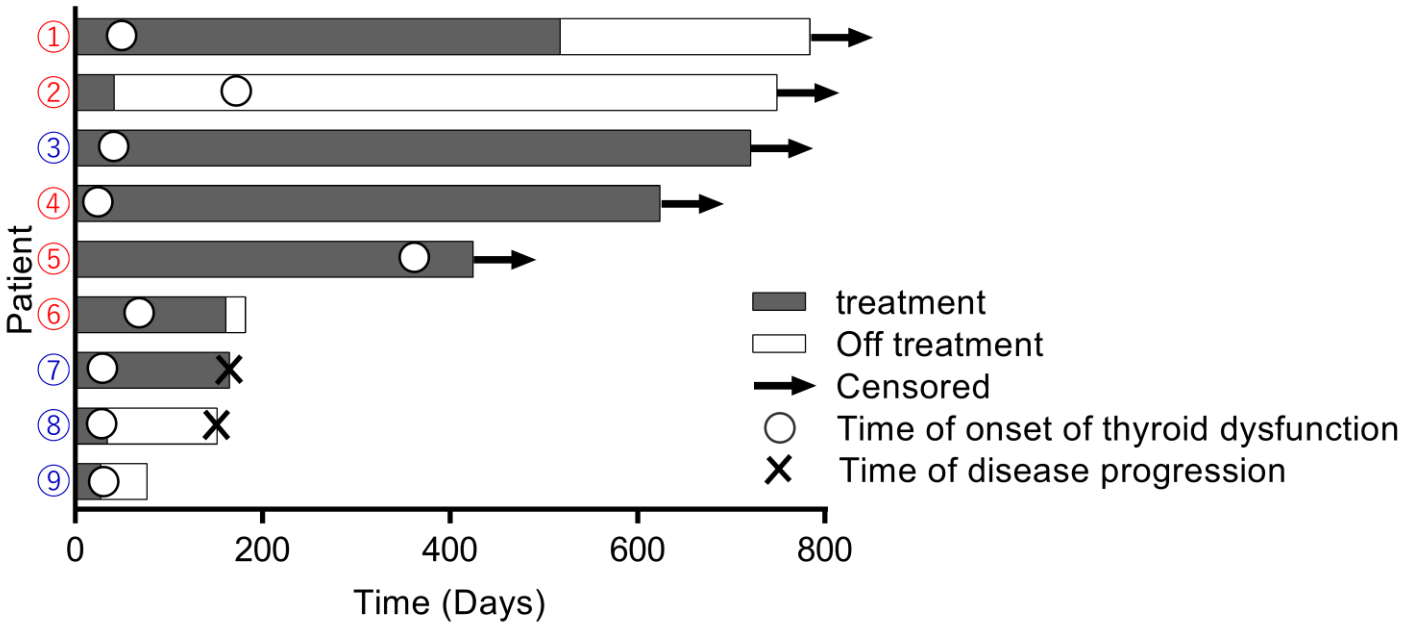

Figure S3. Progression-free free survival (PFS) for patients with low free T4. The number of patients is consistent with that presented in Supplementary Table S3. The lengths of the bars indicate the time between nivolumab initiation and progression in patients with low free T4. The median duration from nivolumab administration to the onset of thyroid dysfunction was 48 days. Two patients (No. 7 and 8) had disease progression and two patients (No. 6 and 9) were switched to another chemotherapy approach before disease progression at the discretion of the physician.

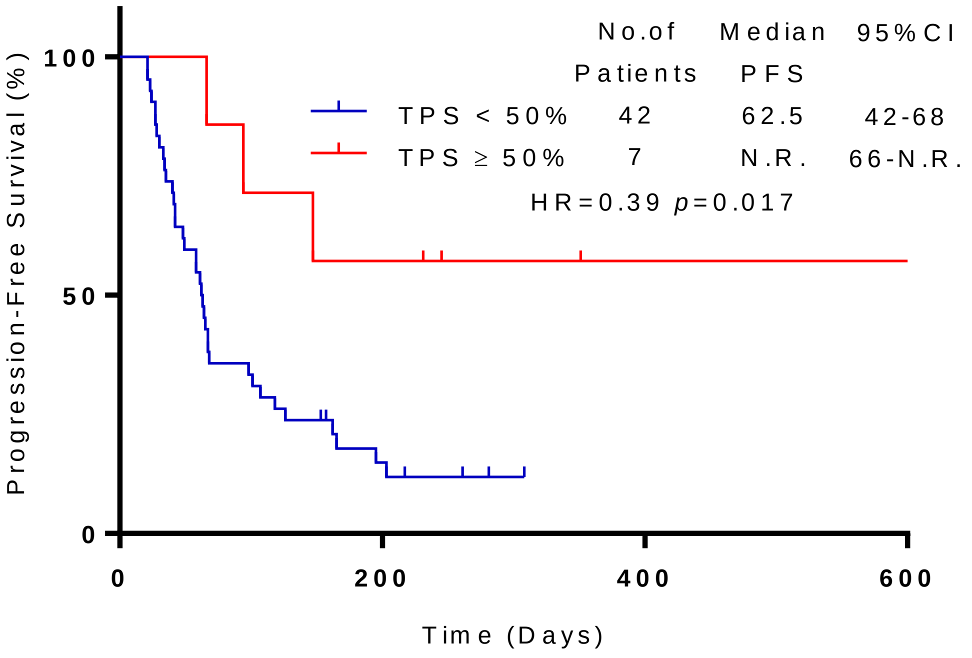

Figure S4. PFS after the administration of nivolumab as shown using the Kaplan–Meier curve. The blue line represents a PD-L1 tumor proportion score (TPS) < 50%, and the red line represents a PD-L1 TPS ≥ 50%.
